# Editorial: Pharmacological and biochemical perspectives of kinase inhibitors in cancer and COVID-19 therapeutics

**DOI:** 10.3389/fphar.2023.1229673

**Published:** 2023-06-06

**Authors:** Balakumar Chandrasekaran, Muthupandian Saravanan

**Affiliations:** ^1^ Faculty of Pharmacy, Philadelphia University, Amman, Jordan; ^2^ AMR and Nanotherapeutics Lab, Department of Pharmacology, Saveetha Dental College, Saveetha Institute of Medical and Technical Sciences (SIMATS), Chennai, Tamilnadu, India

**Keywords:** COVID-19, kinase inhibitors, cancer, hepatocellular carcinoma, JAKs, FDA

On 11th March-2020, World Health Organization (WHO) acknowledged coronavirus disease-2019 (COVID-19) as a pandemic disease that is caused by a severe acute respiratory syndrome coronavirus-2 (SARS-CoV-2). Specifically, COVID-19 is recognized as the third public health crisis suffered by humans around the world accounting for 650 million confirmed cases of infections, besides 6.6 million deaths due to COVID-19 and its associated complications. During the pandemic period of COVID-19, the diagnosis, care, and therapy for cancer patients were significantly affected at large. COVID-19-induced deaths are mostly detected with severe systemic infection among the elderly and immunosuppressed patients including cancer patients. Several cancer patients died due to COVID-19 and the highest number of deaths was reported during the peak of COVID-19 infections (Ali et al., 2022). A research study reported about 1/3 of patients experienced disruptions to cancer care due to the pandemic COVID-19 (Lang et al., 2023). COVID-19 has influenced negatively the progression of the research work pertaining to the clinical trial studies associated with cancer patients. The world is invaded by COVID-19, and there is an urgent need for the proper therapeutic systems to be established apart from the vaccination that took place over the past 3 years (Rucinska and Nawrocki, 2022; Wells and Galvani, 2022).

Kinases are a group of enzymes involved in the transfer of a phosphate group from the phosphate-releasing biomolecules to the specific substrate. They are implicated in varied cellular events such as cell signalling, transport, protein regulation, and metabolism (Chandrasekaran and Saravanan, 2022). Kinases are the most investigated protein targets for the novel design of various anticancer drugs (Yadav, 2022). Amongst, Janus kinases (JAKs) are the crucial mediators in the cytokine storm. Specifically, four JAK isoforms (JAK1, JAK2, JAK3, and TYK2) are involved in transmitting the intracellular signals in corroboration with different types of STATs. Thus, they are considered to be attractive drug targets for novel anticancer drugs including anti-COVID agents (Tanaka, 2023).

This Research Topic is a concise collection of original research articles and review articles pertaining to the computer-aided design, synthesis and pharmacology of novel small molecule inhibitors to be effectively employed for treating cancer and COVID-19. Six impactful articles got published on this Research Topic, *Pharmacological and biochemical perspectives of kinase inhibitors in cancer and COVID-19 therapeutics, Volume II*.

The Research Topic was initiated with a research article titled, “*Thiosemicarbazones and selected tyrosine kinase inhibitors synergize in pediatric solid tumors: NDRG1 upregulation and impaired prosurvival signaling in neuroblastoma cells*” by Krchniakova et al. This research work presented the investigational study on the synergistic properties of three potential kinase inhibitors such as sunitinib, gefitinib, and lapatinib in different combinations with suitable iron-chelating agents Dp44mT or DpC towards controlling solid tumors in paediatric patients. The outcome of this research concluded that the upregulation of a Protein Coding gene NDRG was detected in the combined treatment involving thiosemicarbazones and gefitinib or lapatinib. This study presented a rationale combination of kinase inhibitors and iron chelators that offered a potential strategy for the chemotherapy of neuroblastoma in pediatrics.

The next article in this Research Topic is a review by Grave et al., entitled, “*The functional role of p38 MAPK pathway in malignant brain tumors.*” The review highlighted the recent information regarding the significant involvement of p38 MAPK pathway in the malignant brain tumorigenesis and progression. The authors have presented a good collection of recent studies paving a pathway to a better perception of the significant role of p38 inhibitors and antibodies targeting the glioblastoma tumor-microenvironment in advanced therapies.

Research by Gautam et al., in their contribution, “*Synthesis and appraisal of dalbergin-loaded PLGA nanoparticles modified with galactose against hepatocellular carcinoma: in-vitro, pharmacokinetic, and in silico studies*,” demonstrated nanoscience-based approach in cancer chemotherapy. The authors prepared the synthetic form of dalbergin (DL) which is one of the naturally occurring chemicals that have been subsequently formulated as PLGA-nanoparticles. The *in vitro* evaluation results suggested that DL-modified nano formulation exhibited better therapeutic effectiveness against hepatocellular carcinoma (HCC). Further, this has been supported by *in vivo* pharmacokinetics and bio-distribution reports and *in silico* evaluation of DL against caspase proteins.

The fourth article in this Research Topic, “*Exploring the chemotherapeutic potential of currently used kinase inhibitors: An update*,” is a review by Naik and Shakya that highlighted the recent advances of kinase inhibitors in cancer chemotherapy. This review described the current FDA-approved kinase inhibitors with their molecular mechanisms, and clinical applications including the potential side effects. Moreover, the authors suggested the probable solutions to overcome various side effects of kinase inhibitors and enlightened the future prospects for the discovery of new kinase inhibitors.

The fifth article in this Research Topic is a review by Jain et al., on “*Therapeutic implications of current Janus kinase inhibitors as anti-COVID agents: A review*.” This article outlined the rationale and concepts for utilizing the Janus inhibition towards COVID-19 treatment. Considerable scientific efforts made by the authors to address the discovery of novel drugs against COVID. Authors enlightened the potential involvement of JAK-STAT signalling events in the pathogenesis of COVID-19 and presented an account for the recently approved Jakinibs. Further, this paper highlighted the applications of Jakinibs along with their limitations making this article is a significant review of crucial insights into Jakinibs as anti-COVID agents.

The final article on this Research Topic is titled, “*Efficacy and safety of apatinib versus sorafenib/placebo in first-line treatment for intermediate and advanced primary liver cancer: A systematic review and meta-analysis*,” by Peng et al., reviewed the safety and efficacy of apatinib over other inhibitors. Further, the authors conducted a meta-analysis of the available data from seven of the reported studies. The outcome of this study demonstrated that apatinib could be an additional option for the treatment of HCC patients.

## References

[B1] Ali M. Wani S. U. D. Masoodi M. H. Khan N. A. Shivakumar H. G. Osmani R. M. A. (2022). Global effect of COVID-19 pandemic on cancer patients and its treatment: A systematic review. Clin. Complement. Med. Pharmacol. 2, 100041. 10.1016/j.ccmp.2022.100041 36377228 PMC9035683

[B2] Chandrasekaran B. Saravanan M. (2022). Editorial: Pharmacological and biochemical perspectives of kinase inhibitors in cancer and COVID-19 therapeutics, Volume I. Front. Pharmacol. 13, 916324. 10.3389/fphar.2022.916324 35784709 PMC9240733

[B3] Lang J. J. Narendrula A. Iyer S. Zanotti K. Sindhwani P. Mossialos E. (2023). Patient-reported disruptions to cancer care during the COVID-19 pandemic: A national cross-sectional study. Cancer Med. 12, 4773–4785. 10.1002/cam4.5270 36207994 PMC9874402

[B4] Rucinska M. Nawrocki S. (2022). COVID-19 pandemic: Impact on cancer patients. Int. J. Environ. Res. Public Health 19, 12470. 10.3390/ijerph191912470 36231769 PMC9564768

[B5] Tanaka Y. (2023). A review of Janus kinase inhibitors for the treatment of Covid-19 pneumonia. Inflamm. Regen. 43, 3. 10.1186/s41232-022-00253-3 36617565 PMC9826760

[B6] Wells C. R. Galvani A. P. (2022). Impact of the COVID-19 pandemic on cancer incidence and mortality. Lancet Public Heal 7, e490–e491. 10.1016/S2468-2667(22)00111-6 PMC9159732 35660207

[B7] Yadav D. K. (2022). Editorial: Kinase inhibitors in cancer therapy. Front. Cell Dev. Biol. 10, 1020297. 10.3389/fcell.2022.1020297 36393866 PMC9660224

